# Colorectal Cancer Biomarker Identification via Joint DNA-Methylation and Transcriptomics Analysis Workflow

**DOI:** 10.3390/genes16060620

**Published:** 2025-05-23

**Authors:** Olajumoke B. Oladapo, Marmar R. Moussa

**Affiliations:** 1 Stephenson School of Biomedical Engineering, University of Oklahoma, Norman, OK 73019, USA; 2 School of Computer Science, University of Oklahoma, Norman, OK 73019, USA

**Keywords:** methylation, bulk RNA sequencing, colorectal cancer, methylation-regulated genes, biomarkers

## Abstract

**Background**: Colorectal cancer (CRC) is a term that refers to the combination of colon and rectal cancer as they are being treated as a single tumor. In CRC, 72% of tumors are colon cancer, while the other 28% represent rectal cancer. CRC is a multifactorial disease caused by both genetic and epigenetic changes in the colon mucosal cells, affecting the oncogenes, DNA repair genes, and tumor suppressor genes. Currently, two DNA methylation-based biomarkers for CRC have received FDA approval: *SEPT9*, used in blood-based screening tests, and a combination of *NDRG4* and *BMP3* for stool-based tests. Although DNA methylation biomarkers have been explored in colorectal cancer (CRC), the identification of robust and clinically valuable biomarkers remains a challenge, particularly for early-stage detection and precancerous lesions. Patients often receive diagnoses at the locally advanced stage, which limits the potential utility of current biomarkers in clinical settings. **Methods**: The datasets used in this study were retrieved from the GEO database, specifically GSE75548 and GSE75546 for rectal cancer and GSE50760 and GSE101764 for colon cancer, summing up to a total of 130 paired samples. These datasets represent expression profiling by array, methylation profiling by genome tiling array, and expression profiling by high-throughput sequencing and include rectal and colon cancer samples paired with adjacent normal tissue samples. Differential analysis was used to identify differentially methylated CPG sites (DMCs) and identify differentially expressed genes (DEGs). **Results**: From the integration of DMCs with DEGs in colorectal cancer, we identified 150 candidates for methylation-regulated genes (MRGs) with two genes common across all cohorts (*GNG7* and *PDX1*) highlighted as candidate biomarkers in CRC. The functional enrichment analysis and protein–protein interactions (PPIs) identified relevant pathways involved in CRC, including the Wnt signaling pathway, extracellular matrix (ECM) organization, among other enriched pathways. **Conclusions**: Our findings show the strength of our in silco computational approach in jointly identifying methylation-regulated biomarkers for colon cancer and highlight several genes and pathways as biomarker candidates for further investigations.

## 1. Introduction

Colorectal cancer (CRC) is a term that refers to the combination of colon and rectal cancers, which are treated as a single tumor. In CRC, 72% of tumors are colon cancer, while the remaining 28% are rectal cancer [1]. Colorectal cancer ranks as the third most prevalent cancer and the second largest cause of mortality, with an anticipated incidence rate exceeding 60% by 2030 [1,2]. The 5-year survival rate for 90% of patients diagnosed with CRC in early and localized stages is significantly higher than the 13.1% rate observed in advanced stages and metastatic cases [3]. Early detection is essential for the survival of patients diagnosed with CRC, and biomarkers are pivotal in its diagnosis and prognosis. However, only a limited number of biomarkers have been integrated into clinical practice, underscoring the necessity to develop additional biomarkers in CRC [4]. Currently, microRNAs, DNA mutations, methylation, proteins encompassing various epigenetic functions, and gut microbiomes are areas investigated for the identification of CRC biomarkers [5].

DNA methylation patterns in normal and tumor-specific cells exhibit markedly distinct profiles, which can facilitate the identification of DNA from tumor samples, hence serving as a promising biomarker [6]. Currently, two DNA methylation biomarkers for colorectal cancer (CRC) have been approved by the FDA: *SEPT9*, utilized in blood screening tests, and a combination of *NDRG4* and *BMP3* for stool tests [7]. Recently, many studies have introduced promising DNA methylation biomarkers for CRC. Shen et al. [7] identified two potential CpG site biomarkers for colorectal cancer: cg13096260 and cg12993163, from 76 pairs of CRC and adjacent normal tissue samples, 348 stool samples, and 136 blood samples. In a similar manner, the Stool ColoDefense test used by Zhao et al. [8] found the DNA methylation of *SEPT9* and *SDC2* as a composite biomarker for CRC. Despite the investigation of DNA methylation biomarkers in CRC, the discovery of reliable and clinically significant biomarkers continues to pose a problem, especially for early-stage detection and precancerous lesions. Current biomarkers frequently exhibit insufficient sensitivity and specificity for early detection, resulting in patients typically being diagnosed at a locally advanced stage, which constrains their potential application in clinical environments [4,9].

This study seeks to fill this gap by utilizing a bioinformatics pipeline to find novel DNA methylation-regulated genes linked to CRC. This study employs the methodology established by Li et al. [10], who identified methylation-regulated genes in varicose vein disease and classified these genes as biomarkers for varicose vein disease alongside their traits which were taken into consideration in their analysis. In this study, we aim to identify methylation-regulated genes in CRC samples by analyzing publicly accessible methylation and expression datasets of CRC for candidate biomarkers that demonstrate consistent epigenetic modifications in CRC samples. The primary objective is to identify biomarkers that may be subsequently validated for their diagnostic and prognostic capabilities in CRC and precancerous lesions.

## 2. Materials and Methods

**Figure 1:**
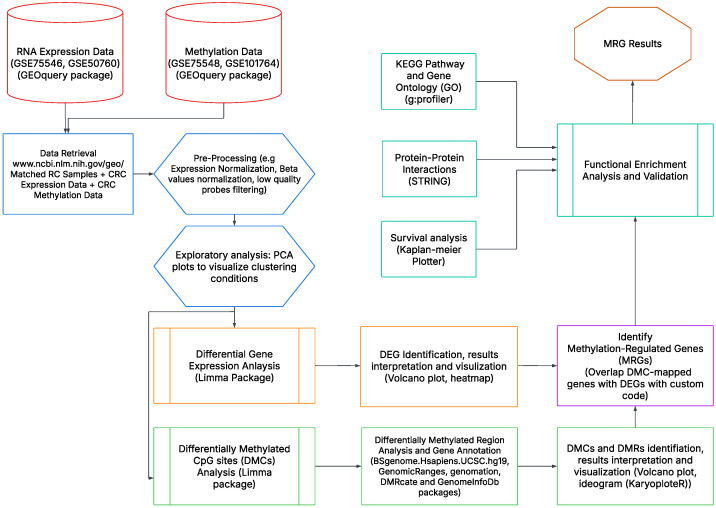
Workflow overview. This workflow illustrates the integrated analysis of matched DNA methylation and RNA expression sample data, incorporating pre-processing, differential analysis, genomic annotation, functional enrichment, and data visualization to identify methylation-regulated genes.

In the following section, we describe the main components of our joint analysis workflow. An overview of the workflow processes is shown in Figure 1.

### 2.1. Data Collection

**Figure 2:**
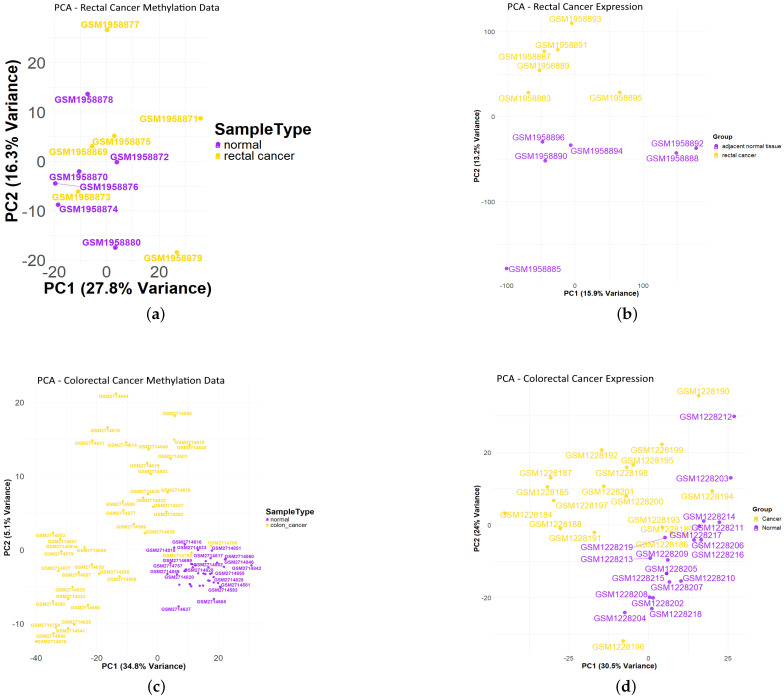
Principal component analysis (PCA) of colorectal cancer samples illustrating distinct clustering patterns between cancerous and normal tissues. Cancer samples exhibit greater dispersion, reflecting heterogeneity, whereas normal samples form a more compact, homogeneous cluster. (**a**) PCA plot of rectal cancer methylation samples. (**b**) PCA plot of rectal cancer expression samples. (**c**) PCA plot of colon cancer methylation samples. (**d**) PCA plot of colon cancer expression samples.

The datasets utilized in this study were retrieved from the Gene Expression Omnibus (GEO) database (https://www.ncbi.nlm.nih.gov/geo/, accessed on 13 May 2025) using the GEOquery package [11]. Specifically, datasets GSE75548 and GSE75546 were obtained for rectal cancer, consisting of matched patient samples. GSE75548 represents expression profiling by microarray, whereas GSE75546 represents methylation profiling by genome tiling array; both datasets contain six paired samples of rectal cancer and corresponding normal tissues. Additionally, datasets GSE50760 (expression profiling by high-throughput sequencing) and GSE101764 (methylation profiling by microarray) were employed for colon cancer analyses. The GSE50760 dataset was subsetted to retain only colon cancer samples, yielding a total of 36 samples. The GSE101764 dataset was filtered to include paired samples from patients aged 40 and above, resulting in a total of 82 samples, thus ensuring consistency in biological characteristics, including age, across all analyses. In total, 130 samples are analyzed in this study; Figure 2 summarizes the samples in both expression and methylation data via principal component analysis.

### 2.2. Identifying and Mapping Differentially Methylated CpG Sites

Differentially methylated CpG sites (DMCs) between normal and cancer tissue samples were identified using the Limma package [12]. For rectal cancer, DMCs with Adj.P.Value < 0.05 and $\log_{2}\mathrm{FC}>1$ were considered statistically significant. Due to the larger sample size available for colon cancer analyses, more stringent thresholds were applied, with significance defined by an Adj.P.Value < 0.01 and $\log_{2}\mathrm{FC}>2$. Differentially methylated regions (DMRs) between normal and cancer samples were identified using the DMRcate package [13], with a false discovery rate (FDR) threshold of 0.001. DMRs were defined as genomic regions containing at least two significant DMCs (C = 2) within a 1000 bp window (λ=1000). Genomic coordinates of identified regions were validated using the BSgenome.Hsapiens.UCSC.hg19 package [14], ensuring the inclusion of only standard autosomal chromosomes. Subsequently, DMCs obtained from Limma were cross-referenced with the DMR results, classifying them into hypermethylated, hypomethylated, or non-significant categories. A karyogram visualizing hypermethylated (red) and hypomethylated (blue) genomic regions was generated using the karyoploteR package [15].

### 2.3. Normalization and Filtering

Expression data for rectal cancer were obtained in a pre-normalized form from the GEO archive and further filtered using median expression values. For colon cancer, the normalization and filtering of samples were performed using EdgeR [16], ensuring minimal batch effect in expression and methylation data (see Supplementary Figure S1).

### 2.4. Identification of Differentially Expressed Genes (DEGs)

Differentially expressed genes (DEGs) between normal and cancer tissue samples were identified using the Limma package [12]. For rectal cancer, DEGs with Adj.P.Value < 0.05 and $\log_{2}\mathrm{FC}>1$ were considered statistically significant. Due to the larger sample size available for colon cancer analyses, more stringent thresholds were applied, with significance defined by an Adj.P.Value < 0.01 and $\log_{2}\mathrm{FC}>2$. The results were visualized using a volcano plot to highlight upregulated, downregulated, and non-significant genes.

### 2.5. Statistical Methods for Differential Analyzes

As discussed in previous sections, Limma R package and algorithms were used to calculate differential expression (or methylation). To summarize, this method apply fitting linear models to normalized expression data, considering factors like inter-gene correlation and precision weights. The method then compares the expression levels of different groups or conditions using *t*-tests, identifying genes with significant differences.

### 2.6. Identification and Analysis of Methylation-Regulated Genes (MRGs)

Gene symbols from the annotated DMRs were compared with significantly differentially expressed genes (DEGs). This integration identified common genes: methylation-regulated genes (MRGs) that showed both methylation alterations and differential expression patterns.

### 2.7. Validation and Functional Enrichment of Methylation-Regulated Genes

Biological processes and pathways associated with methylation-regulated genes (MRGs) were identified using Gene Ontology (GO) and KEGG pathway enrichment analysis performed using g:Profiler [17] methods. Protein–protein interaction (PPI) networks were constructed using the STRING database [18] to identify gene clusters and their associated functional and regulatory pathways, further validating the methylation-based regulation of genes. Additionally, survival analysis (Kaplan–Meier (KM) [19] overall survival (OS) method) was conducted on select genes using clinical data from colon and rectal cancer patients to evaluate the prognostic significance of two spotlight MRGs.

## 3. Results

### 3.1. Differentially Methylated CpG Sites (DMCs) and Differentially Expressed Genes (DEGs) of Rectal Cancer Cohort

**Figure 3:**
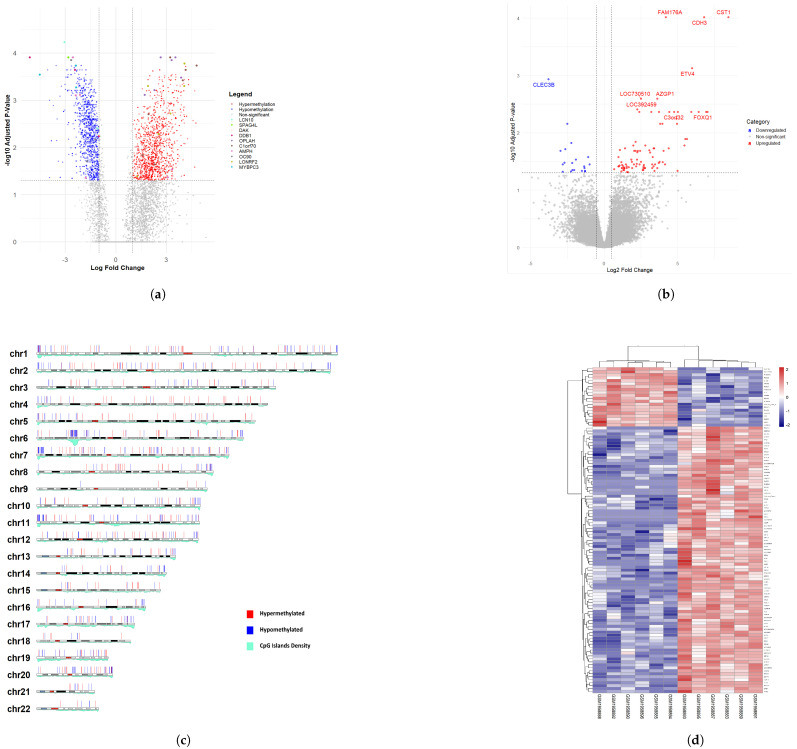
Combined visualization of rectal cancer methylation and expression analyses. (**a**) Volcano plot of rectal cancer methylation analysis. (**b**) Volcano plot of rectal cancer expression analysis. (**c**) Karyogram showing DMRs with significant methylation changes. (**d**) Heatmap of the top 50 differentially expressed genes.

The methylation and expression datasets of rectal cancer were analyzed to identify differentially methylated CpG sites (DMCs) by fitting a generalized linear model from limma. The results from the DMC analysis were cross-referenced with differentially methylated regions (DMRs) to enhance the reliability of the findings. A total of 678 genes were classified as significantly hypermethylated or hypomethylated within the identified DMRs. Differential expression analysis revealed 101 genes that were significantly up- or downregulated in rectal cancer. The lists of significant genes from the methylation and expression analyses are provided in Supplementary Tables S1 and S2, respectively. Figure 3 illustrates the volcano plots for DMCs and DEGs, the karyogram highlighting DMRs with significant hypermethylation and hypomethylation, and a heatmap of the top 50 differentially expressed genes.

### 3.2. Differentially Methylated CpG Sites (DMCs) and Differentially Expressed Genes (DEGs) of Colon Cancer Cohort

**Figure 4:**
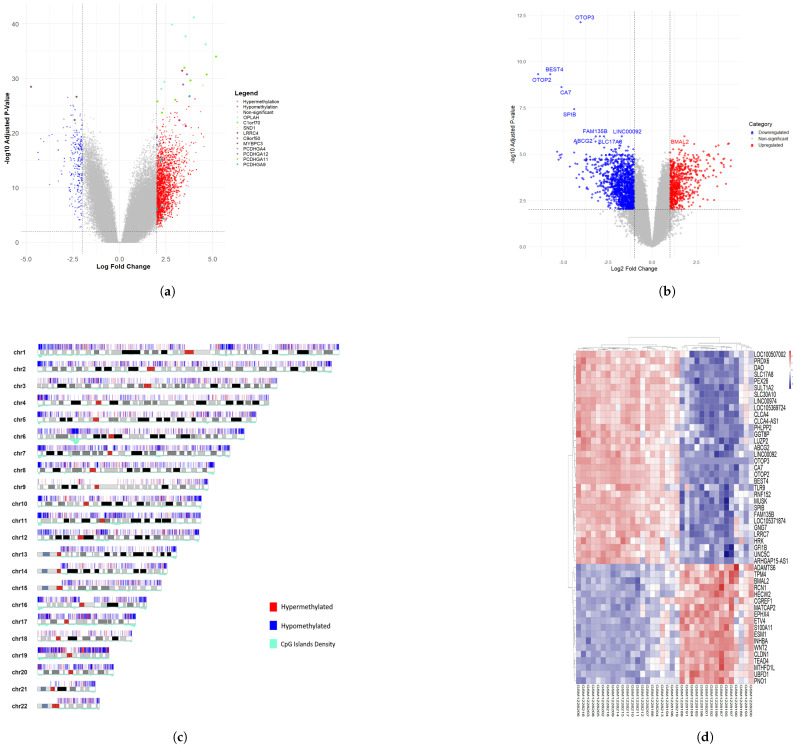
Combined visualization of colon cancer methylation and expression analyses. (**a**) Volcano plot of colon cancer methylation analysis. (**b**) Volcano plot of colon cancer expression analysis. (**c**) Karyogram showing DMRs with significant methylation changes. (**d**) Heatmap of the top 50 differentially expressed genes.

Conserving the methodology applied to the rectal cancer cohort, we extended this analysis to the colon cancer datasets. The methylation and expression data of the colon cancer samples were analyzed to identify differentially methylated CpG sites (DMCs) by fitting a linear model from limma. The results from the DMC analysis were cross-referenced with differentially methylated regions (DMRs) to enhance the reliability of the findings. A total of 1053 genes were classified as significantly hypermethylated or hypomethylated within the identified DMRs. Differential expression analysis revealed 2130 genes that were significantly upregulated or downregulated in the colon cancer group. The lists of significant genes from the methylation and expression analyses are provided in Supplementary Tables S3 and S4, respectively. Figure 4 illustrates the volcano plots for DMCs and DEGs, the karyogram highlighting DMRs with significant hypermethylation and hypomethylation, and a heatmap of the top 50 differentially expressed genes.

### 3.3. Methylation-Regulated Genes (MRGs)

Out of the 678 unique genes identified from both the DMC and DMR analyses in rectal cancer, six genes that overlapped with the 101 differentially expressed genes (DEGs). Similarly, 146 overlapping genes were identified from the colon cancer DMC and DMR analyses with corresponding DEGs. In total, 150 genes were inferred as methylation-regulated genes (MRGs) across the total colorectal cancer cohort, with two genes in particular—*PDX1* and *GNG7*—consistently identified in both rectal and colon cancer individual analyses. These common genes were considered as promising candidates of MRGs. To validate the identified MRGs, we conducted further functional annotation, pathway enrichment, and survival analysis, highlighting the role of these genes in CRC. A select group of the identified MRGs, along with their $\log_{2}$ fold change, average expression, and adjusted *p*-values, is summarized in Table 1, which highlight all genes pertaining to rectal cancer cohort (six genes in total) and the top 10 MRGs from colon cancer; the full list includes the shared genes (highlighted in bold) across all samples.

### 3.4. Validation and Functional Enrichment of Methylation-Regulated Genes

**Figure 5:**
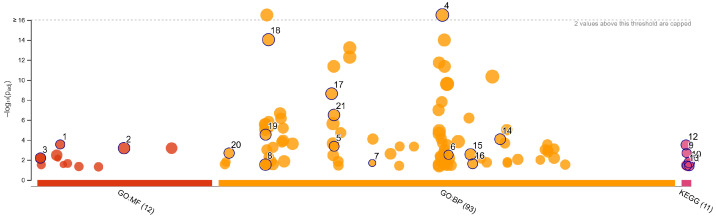
Pathway enrichment analysis of methylation-regulated genes.

The methylation-regulated genes (MRGs) were further subjected to functional enrichment analysis using KEGG and Gene Ontology databases via g:Profiler methods [19]. Key biological pathways identified from the enrichment results include the Wnt signaling pathway, pathways in cancer, and extracellular matrix organization, among others. Additionally, several neurogenesis and neuron development pathways were identified, highlighting the role of the nervous system (enteric nervous system) in the etiology and development in CRC. Figure 5 presents the enrichment and functional analysis results, highlighting the top enriched pathways associated with MRGs. Table 2 summarizes the functional pathways associated with the methylation-regulated genes (MRGs), as identified through Gene Ontology and KEGG pathway enrichment analysis.

**Figure 6:**
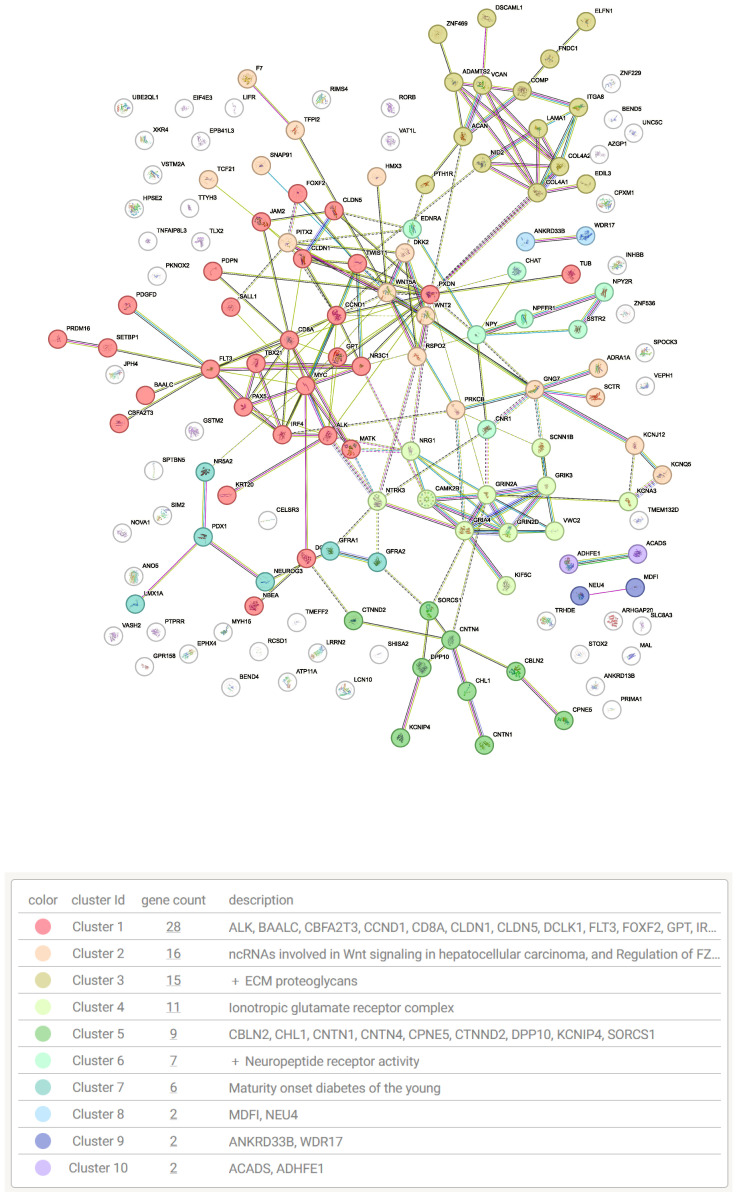
Gene network analysis highlighting relevant gene clusters and pathways.

In addition, Figure 6 illustrates the protein–protein interaction (PPI) network generated using the STRING database, along with functionally relevant pathways derived from these interactions.

**Figure 7:**
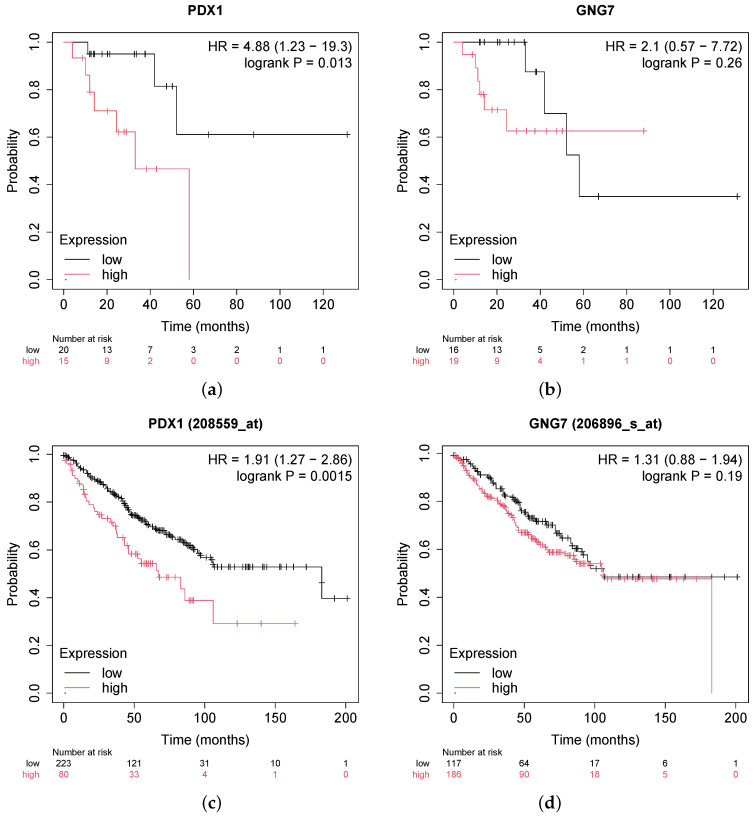
Kaplan Meier Survival Analysis. (**a**) Survival analysis of *PDX1* expression level across cohorts with rectal cancer (**b**) Survival analysis of *GNG1* expression level across cohorts with rectal cancer (**c**) Survival analysis of *PDX1* expression level across cohorts with colon cancer (**d**) Survival analysis of *GNG7* expression level across cohorts with colon cancer.

Furthermore, we performed additional validation through survival analysis performed on public clinical data for two selected highlighted genes, *PDX1* and *GNG7*—which were commonly identified in both datasets. This analysis is presented in Figure 7. These Kaplan–Meier plots illustrate the association between gene expression levels and patient survival across rectal and colon cancer cohorts.

## 4. Discussion

Colorectal cancer (CRC) arises when the normal epithelial cells of the colon and rectum undergo transformation into a precancerous lesion, ultimately progressing to an advanced carcinoma capable of metastasizing to other organs [1]. The risks of developing colorectal cancer (CRC) are associated with age, environmental influences, behavioral patterns, and genetic determinants [20]. Raut et al. [21] identified two fecal DNA methylation biomarkers for detecting stages in colorectal cancer (CRC). Bach et al. [22] discovered *SEPT9* and *SDC2* as critical markers for non-invasive colorectal cancer (CRC) detection by urine-based DNA methylation analysis. DNA methylation has been extensively studied in CRC; Huang et al. [23] identified distinct tumor clusters with methylated CpG islands linked to metabolic pathways, enhanced ATP production, and tumor aggressiveness in CRC.

In this current study, we analyzed data from a publicly available dataset on colon and rectal cancer samples and carried out differential methylation and expression analysis on these datasets. We identified significant hypermethylated and hypomethylated genes in CRC and found genes that were methylation-regulated suggesting methylation plays a role in the alterations of these gene expression patterns. Similarly, Miao et al. [24], through an integrated analysis in the pathogenesis of coronary artery disease, found overlaps between differentially methylated genes (DMGs) and DEGs through their intersection and carried out subsequent analysis to highlight genes important in the pathogenesis of coronary heart disease. Sun et al. [25], through an integrated analysis, identified eight genes that are regulated by methylation and proposed these genes to have therapeutic and diagnostic relevance in lung cancer.

A total of 150 genes were identified as MRGs from CRC analysis which includes *PDX1* and *GNG7* as spotlight genes were consistently found in rectal as well as colorectal cancer samples in both differentially expressed and methylated gene groups.

Findings from Liu et al. [26] showed 411 upregulated genes that were significantly hypomethylated and 239 downregulated genes that were hypermethylated. The hub genes that can serve as important biomarkers for CRC. Similarly, Sun et al. [27] identified hub genes that were differentially expressed in CRC analysis and suggested these hub genes as biomarkers of CRC.

In this study, we identified 101 and 2130 significant differentially expressed genes (DEGs) in rectal and colon cancer, respectively. Correspondingly, 678 and 1053 significant differentially methylated CpG sites (DMCs) were detected in rectal and colon cancer. By intersecting the DEGs and DMCs from each dataset, we identified a total of 150 methylation-regulated genes (MRGs). Notably, *PDX1* and *GNG7* were common to both rectal and colon cancer analyses, with *GNG7* also ranking among the top ten genes in colon cancer.

*GNG7*, a component of heterotrimeric G proteins, is highly enriched in the striatum and plays a crucial role in the neuroprotective response mediated by A2A adenosine and D1 dopamine receptors. Previous studies have reported *GNG7* downregulation in various cancers, including pancreatic, gastrointestinal tract, renal, and lung cancers [28]. In our study, we identified *GNG7* as being downregulated and hypomethylated in colorectal cancer. *PDX1* is predominantly expressed in the islets of Langerhans, central nervous system, and gastrointestinal tract [29,30]. It is a critical transcription factor involved in pancreas development and has been implicated in colorectal cancer (CRC). A recent study by Lee et al. [31] reported that the hypermethylation of *PDX1* serves as a potential biomarker for CRC prognosis [31]. Consistent with these findings, our current analysis also demonstrates that *PDX1* is hypermethylated and correspondingly upregulated in colorectal cancer samples.

We performed KEGG pathway enrichment and Gene Ontology (GO) analyses using g:Profiler, alongside protein–protein interaction (PPI) and gene network enrichment analyses via the STRING database, to explore the functional significance of the identified methylation-regulated genes (MRGs). Important pathways enriched among the MRGs include the Wnt signaling pathway, extracellular matrix (ECM) organization, neurogenesis and neuronal differentiation, and maturity-onset diabetes of the young. Zhu et al. [32] reported that the Wnt signaling pathway plays a crucial role in colorectal cancer (CRC), particularly affecting the survival and proliferation of CRC cells, including cancer stem cells. Similarly, Li et al. [33] highlighted that genetic aberrations in components of the Wnt/β-catenin signaling pathway are associated with CRC progression. Karlsson et al. [34] identified the ECM as a potential prognostic marker for CRC due to its critical role within the tumor microenvironment and its possible contribution to metastasis. In agreement, Kim et al. [35] also suggested ECM components as important biomarkers for CRC. Additionally, our PPI network analysis highlighted genes implicated in neurogenesis and neural differentiation. Gut autonomic functions are regulated by the enteric nervous system, and impairments in this system could disrupt interactions with other cellular components, potentially driving CRC tumorigenesis [36,37,38]. Several studies have reported associations between colorectal cancer and Type II diabetes mellitus (T2DM). Liu et al. [39] reviewed evidence demonstrating increased DNA methylation at multiple CpG sites in pancreatic islets of T2DM patients, which significantly reduces *PDX1* mRNA expression, impairing insulin secretion. Similarly, Cheng et al. [40] reviewed how insulin resistance might influence tumor growth, thereby linking diabetes and colorectal cancer progression. Survival analysis was carried out for the two spotlight genes, and the high expression of *PDX1* was seen to be correlated to low survival, while *GNG7* upregulation and downregulation showed similar low survival across samples.

In conclusion, 150 genes were identified as methylation-regulated genes through a comprehensive bioinformatics analysis, suggesting that methylation affects their expression levels. These genes have been associated with a variety of tumors in literature studies, with some specifically linked to colorectal cancer (CRC). We propose the highlighted genes could serve as biomarkers for CRC etiology and disease prognosis. Our study is limited to the secondary analysis, and further experimental tests can further validate the functional insights gained from this study. We look forward to continuing experimental validation as a future direction for this project.

## Figures and Tables

**Table 1:** Methylation Regulated genes identified from combined colorectal cancer analysis.

RC MRGs								
**Gene**	**logFC (Expr)**	**AveExpr (Expr)**	**t (Expr)**	***p*** **.Value (Expr)**	**Adj.P.Val (Expr)**	**B (Expr)**	**Up- or Downregulation**	**Hyper- or Hypomethylation**
*GNG7*	−2.02633	4.84152	−5.95184	$9.20\times 10^{-5}$	0.04456	1.72179	Down	Hypo
*HKDC1*	3.60053	5.90876	6.04456	$8.04\times 10^{-5}$	0.04150	1.84160	Up	Hypo
*AZGP1*	3.60273	2.10376	10.7325	$3.33\times 10^{-7}$	0.00255	6.29499	Up	Hypo
*ALG1L*	3.05863	1.81574	7.23563	$1.58\times 10^{-5}$	0.02028	3.25665	Up	Hypo
*PITX2*	3.70735	2.94234	6.50273	$4.21\times 10^{-5}$	0.03376	2.41281	Up	Hyper
*PDX1*	4.37360	3.77735	7.36968	$1.33\times 10^{-5}$	0.01871	3.40209	Up	Hyper
**CRC MRGs**								
*WNT2*	4.33366	−0.08931	7.87975	$2.10\times 10^{-9}$	$2.69\times 10^{-6}$	11.45417	Up	Hyper
*UNC5C*	−1.79796	2.91492	−7.67632	$3.86\times 10^{-9}$	$3.52\times 10^{-6}$	10.87752	Down	Hyper
*CLDN1*	3.60033	2.97942	7.62796	$4.46\times 10^{-9}$	$3.88\times 10^{-6}$	10.73974	Up	Hypo
*GNG7*	−2.20229	2.97970	−7.24621	$1.41\times 10^{-8}$	$7.11\times 10^{-6}$	9.64326	Down	Hypo
*EPHX4*	2.89609	−0.17779	7.23503	$1.46\times 10^{-8}$	$7.16\times 10^{-6}$	9.61091	Up	Hypo
*PDPN*	2.03958	3.26737	6.94714	$3.50\times 10^{-8}$	$1.10\times 10^{-5}$	8.77430	Up	Hyper
*TRHDE*	−1.74839	1.34963	−6.77779	$5.88\times 10^{-8}$	$1.33\times 10^{-5}$	8.27887	Down	Hyper
*CPNE5*	−1.31592	3.69191	−6.64090	$8.95\times 10^{-8}$	$1.67\times 10^{-5}$	7.87689	Down	Hyper
*VWC2*	−1.58839	−0.61997	−6.47318	$1.50\times 10^{-7}$	$2.00\times 10^{-5}$	7.38269	Down	Hyper
*COL4A1*	2.22504	7.58071	6.44237	$1.65\times 10^{-7}$	$2.01\times 10^{-5}$	7.29174	Up	Hyper

**Table 2:** Functional pathways of MRGs.

Source	Term Name	Term ID	−log10 p	Intersect
GO:MF	extracellular matrix structural constituent	GO:0005201	3.5524	10
GO:MF	signaling receptor activity	GO:0038023	3.1813	29
GO:MF	DNA-binding transcription activator activity...	GO:0001228	2.1923	14
GO:BP	system development	GO:0048731	16.3616	75
GO:BP	nervous system development	GO:0007399	14.0332	57
GO:BP	neurogenesis	GO:0022008	8.6293	40
GO:BP	neuron differentiation	GO:0030182	6.5024	33
GO:BP	enzyme-linked receptor protein signaling pathway	GO:0007167	4.5302	24
GO:BP	response to growth factor	GO:0070848	4.0768	20
GO:BP	extracellular matrix organization	GO:0030198	3.3660	13
GO:BP	morphogenesis of an epithelium	GO:0002009	2.6869	15
GO:BP	epithelium development	GO:0060429	2.5588	24
GO:BP	neuromuscular process	GO:0050905	2.5201	9
GO:BP	ionotropic glutamate receptor signaling pathway	GO:0035235	1.6912	4
GO:BP	epithelial tube morphogenesis	GO:0060562	1.6144	11
GO:BP	cell adhesion	GO:0007155	1.5264	25
KEGG	Neuroactive ligand-receptor interaction	KEGG:04080	3.5077	14
KEGG	Wnt signaling pathway	KEGG:04310	2.6935	9
KEGG	Pathways in cancer	KEGG:05200	1.7382	14
KEGG	Cell adhesion molecules	KEGG:04514	1.4320	7
KEGG	Proteoglycans in cancer	KEGG:05205	1.3830	8

## Data Availability

The data used in this work are publicly available from GEO archive under accession number GSE75550, GSE50760 and GSE101764.

## Supplementary Materials

The following supporting information can be downloaded at: https://www.mdpi.com/article/10.3390/genes16060620/s1. Table S1: rectal cancer methylation DMC; Table S2: rectal cancer expression DEG; Table S3: colon cancer methylation DMC; Table S4: colon cancer expression DEG; Figure S1: Normalization/Batch Effect; Figure S2: Venn diagram showing the gene overlap in colon and rectal cancer

## Abbreviations

The following abbreviations are used in this manuscript:

CRC	Colorectal cancer
MRGs	Methylation-regulated genes
DMCs	Differentially methylated CpG sites
DMRs	Differentially methylated regions
DEGs	Differentially expressed genes
