# Supplementary material for: Colorectal Cancer Biomarker Identification via Joint DNA-Methylation and Transcriptomics Analysis Workflow
Source: Genes (Basel). 2025 May 23;16(6):620. doi: 10.3390/genes16060620 (PMC12191883; doi:10.3390/genes16060620)
Supplement: Supplementary file 1 [file genes-16-00620-s001.zip › Colorectal_cancer Supplementary Materials.pdf]

Figure S1: Normalization/Batch Effect

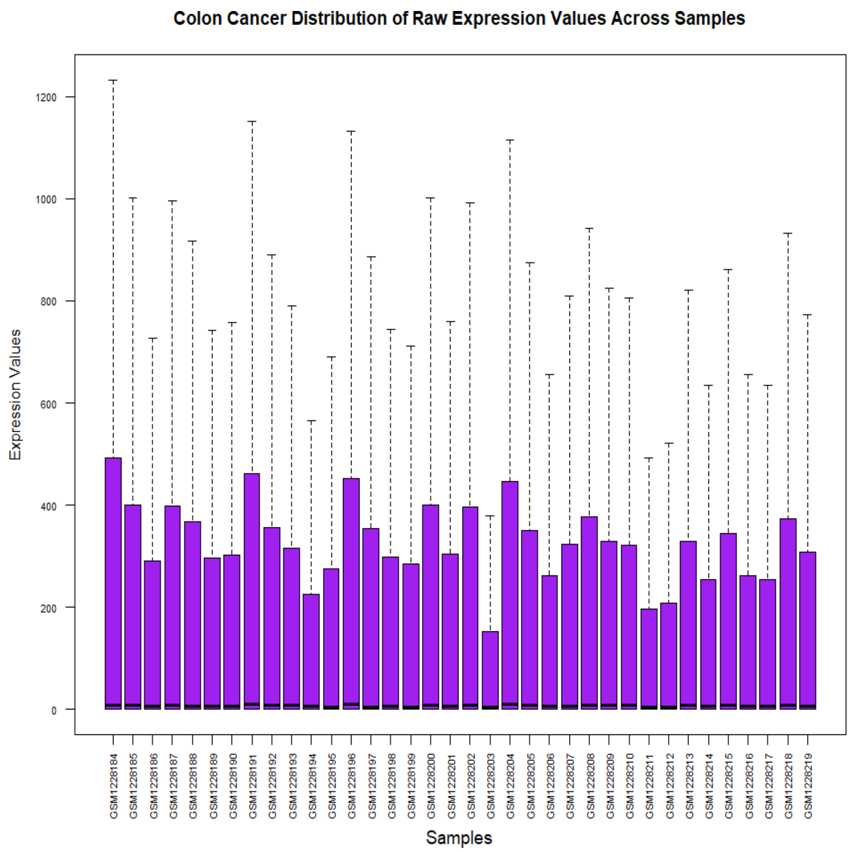

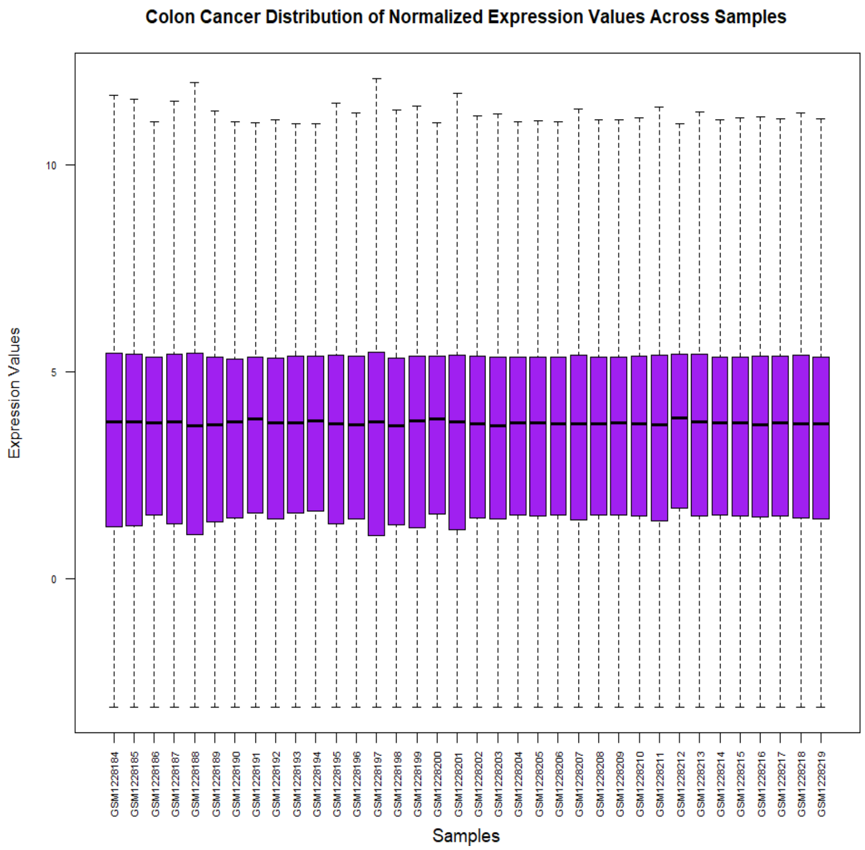
Colon Cancer Distribution of Normalized Expression Values Across Samples
Expression Values
10
5
0
GSM1228184
GSM1228185
GSM1228186
GSM1228187
GSM1228188
GSM1228189
GSM1228190
GSM1228191
GSM1228192
GSM1228193
GSM1228194
GSM1228195
GSM1228196
GSM1228197
GSM1228198
GSM1228199
GSM1228200
GSM1228201
GSM1228202
GSM1228203
GSM1228204
GSM1228205
GSM1228206
GSM1228207
GSM1228208
GSM1228209
GSM1228210
GSM1228211
GSM1228212
GSM1228213
GSM1228214
GSM1228215
GSM1228216
GSM1228217
GSM1228218
GSM1228219
Samples

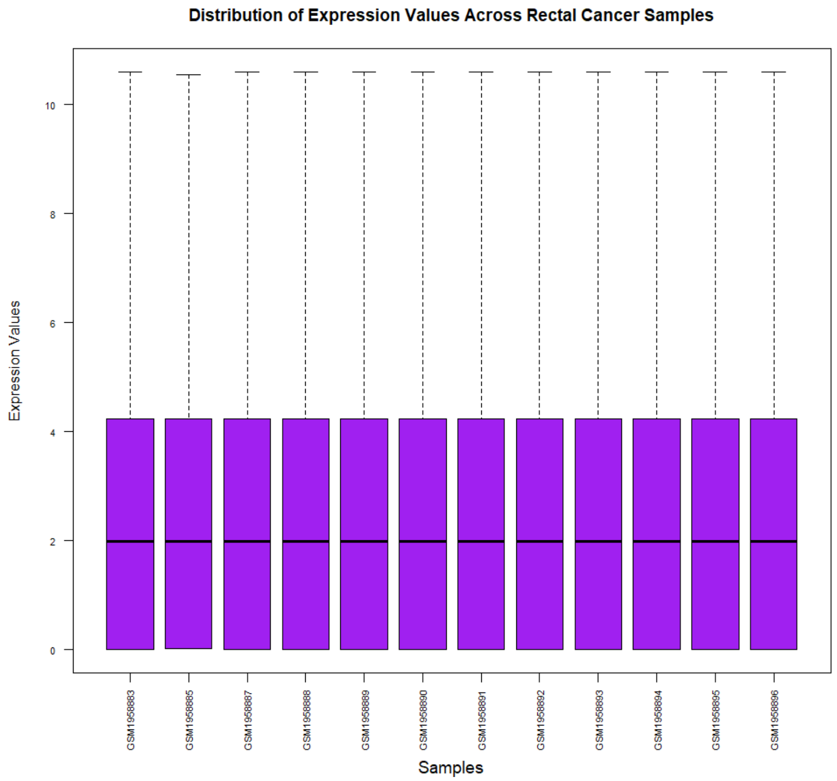
Distribution of Expression Values Across Rectal Cancer Samples
Expression Values
0
2
4
6
8
10
GSM1958883
GSM1958885
GSM1958887
GSM1958888
GSM1958889
GSM1958890
GSM1958891
GSM1958892
GSM1958893
GSM1958894
GSM1958895
GSM1958896
Samples

Figure S2: Venn diagram showing the gene overlap in colon and rectal cancer

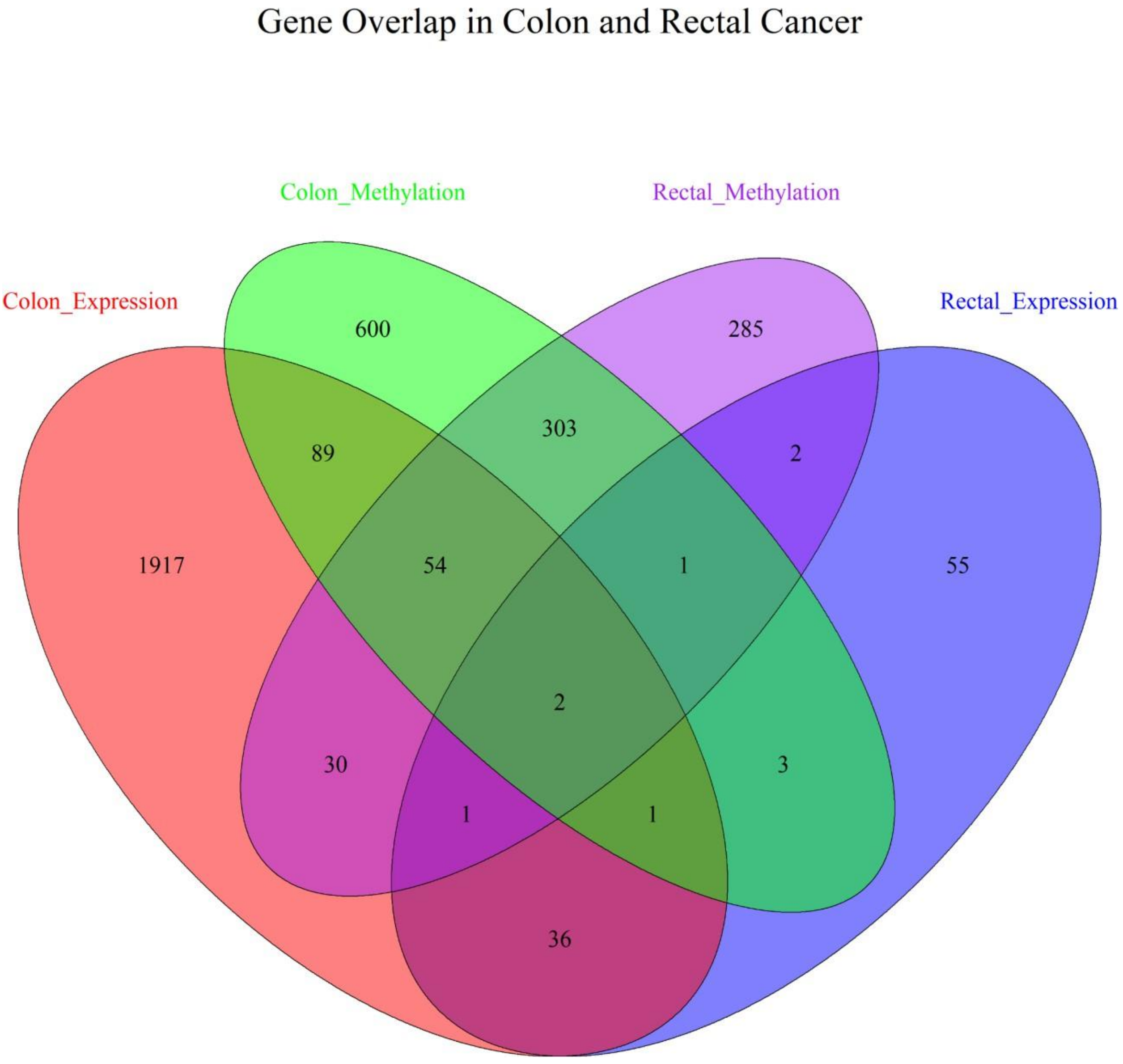

MRG full List:

NR5A2

GRIN2D

ADHFE1

PDX1

LCN10

NPFFR1

FNDC1

CNR1

VWC2

MYH15

TLX2

ANKRD13B

TMEM132D

CBFA2T3

RIMS4

SIM2

CLDN1

NEUROG3

NPY

SNAP91

IRF4

ARHGAP20

PXDN

NR3C1

MDFI

GRIA4

ITGA8

DPP10

MAL

CBLN2

F7

SORCS1

CHAT

HPSE2

COL4A1

COL4A2

PRDM16

GNG7

PRKCB

SETBP1

SPTBN5

UNC5C

LIFR

LMX1A

RSPO2

CNTN4

STOX2

CTNND2

ZNF229

FOXF2

KCNQ5

BEND5

KCNA3

TWIST1

GSTM2

CD8A

WDR17

ELFN1

SALL1

BEND4

TFPI2

SCNN1B

PDGFD

CNTN1

KRT20

GFRA1

PKNOX2

MYC

NBEA

DKK2

CAMK2B

SLC8A3

JAM2

TRHDE

GRIN2A

NPY2R

LRRN2

ADAMTS2

CELSR3

NOVA1

ACAN

JPH4

CPXM1

CCND1

ZNF469

DCLK1

GRIK3

MATK

PDPN

CPNE5

NTRK3

EIF4E3

SCTR

PAX5

TMEFF2

TCF21

NRG1

ATP11A

LAMA1

GPR158

ZNF536

PRIMA1

GFRA2

TNFAIP8L3

HMX3

EDIL3

CLDN5

EPB41L3

ADRA1A

CHL1

WNT2

BAALC

SPOCK3

KCNIP4

TUB

WNT5A

ALK

VAT1L

SHISA2

VCAN

ACADS

NID2

COMP

VEPH1

EPHX4

TTYH3

KIF5C

RORB

VSTM2A

RCSD1

VASH2

FLT3

INHBB

TBX21

ANKRD33B

KCNJ12

PTPRR

UBE2QL1

SSTR2

ANO5

XKR4

GPT

DSCAML1

NEU4

EDNRA

PTH1R

AZGP1

PITX2

ALG1L

HKDC1
